# Multidisciplinary corroboration in differentiation syndrome: a case report

**DOI:** 10.1186/s13256-021-02790-w

**Published:** 2021-05-05

**Authors:** Sayan Sarkar, Priya Ghosh, Anisha Gehani, Niharendu Ghara, Parthasarathi Bhattacharyya

**Affiliations:** 1 Department of Radiology, Tata Medical Center, 14 MAR (E-W), New Town, Rajarhat, Kolkata, 700160 West Bengal India; 2 Department of Pediatric Haematology, Tata Medical Center, 14 MAR (E-W), New Town, Rajarhat, Kolkata, 700160 West Bengal India; 3 Department of Pediatric ICU, Tata Medical Center, 14 MAR (E-W), New Town, Rajarhat, Kolkata, 700160 West Bengal India

**Keywords:** Differentiation, Syndrome, Lung, Leukaemia, ATRA, ATO

## Abstract

**Background:**

Differentiation syndrome (DS) is a life-threatening complication that may be seen in patients with acute promyelocytic leukaemia undergoing induction therapy with all-trans retinoic acid or arsenic trioxide. It can lead to severe inflammatory response syndrome and shock if adequate measures are not taken immediately. The radiological features of lung nodules with changes in ground-glass opacity can represent DS. The principal unique feature of the case reported here is that the diagnosis of DS was based on imaging results in the absence of a low total leukocyte count.

**Case presentation:**

A 14-year-old Indian girl diagnosed with acute promyelocytic leukaemia currently undergoing a chemotherapy regimen that included all-trans retinoic acid/arsenic trioxide was sent to the radiology department for investigation of respiratory distress which she had developed soon after the initiation of chemotherapy. Her chest radiograph showed bilateral lower zone lung infiltrates. Computed tomography (CT) revealed changes in ground-glass opacity in the lower lobes with multiple lung nodules. Differential diagnosis included bacterial, viral or fungal infections, leukemic infiltrates, drug toxicity, pulmonary haemorrhage or leukostasis. She was started on dexamethasone immediately after stopping the chemotherapy with all-trans retinoic acid/arsenic trioxide and given ventilatory support. Her condition subsequently improved and her follow-up chest radiograph and CT scan showed a significant reduction of abnormal lung findings. Based on the clinical improvement and the resolution of findings on imaging following the withdrawal of all-trans retinoic acid/arsenic trioxide, we made the diagnosis of DS.

**Conclusions:**

Though a rather unusual possibility, the treatment history of the patient enabled a rather crucial diagnosis in the nick of time and imaging played a pivotal role. This case further iterates the importance of keeping DS in mind when dealing with similar patients in the future.

## Background

Acute promyelocytic leukaemia (APML) is a subtype of acute myelocytic leukaemia (AML) that usually occurs in patients aged < 40 years; it accounts for approximately 10–15% of all AML cases [1]. APML is characterised by the specifc chromosomal translocation t(15;17). Differentiation syndrome (DS) is seen when patients with APML are treated with all-trans retinoic acid (ATRA) or arsenic trioxide (ATO). Presenting symptoms are varied but frequently include dyspnoea, unexplained fever, unusual weight gain, unexplained hypotension, acute renal failure and a chest radiograph demonstrating pulmonary infiltrates or pleural or pericardial effusion [2].

ATRA belongs to a class of chemical compounds related to vitamin A that are also known as retinoids. These were first introduced in the treatment regimen of APML in the 1980s and have since revolutionised the concept of cancer treatment by not only destroying pathological cells but also supporting the completion of disrupted maturation. Treatment of APML with ATRA/ATO has improved remission rates to 90% and cure rates to approximately 80%, in comparison to rates of < 20% before its use [3]. The clinical picture of DS may be due to cellular migration, endothelial activation, release of interleukins and vascular factors responsible for tissue damage. Although a “cytokine storm” has been described to coincide with the differentiation of blasts, the exact underlying etiopathogenic mechanisms of DS remain partially unknown. The diagnosis of DS is mainly based on clinical and radiological features in the context of induction therapy of APML with differentiating agents, and it usually requires the exclusion of alternative causes that could explain the signs and symptoms of the syndrome [4].The radiological features are pulmonary nodules usually accompanied by changes in ground-glass opacity.

The radiological features can be easily mistaken for other causes if proper vigilance is not maintained. Laboratory evaluations frequently reveal leucocytosis [defined as a white blood cell count (WBC) > 10 × 10^9^/l] and coagulopathy (prolongation of the partial thromboplastin time, prothrombin time and decreased fibrinogen) in patients with APML receiving differentiation therapy. About half of the patients treated with ATRA + ATO will develop leucocytosis compared to one-quarter of those receiving ATRA + chemotherapy [2]. Regarding the incidence of DS, most studies have reported that approximately 25% of patients with APML receiving ATRA as induction therapy develop this syndrome, but the incidence depends on the criteria employed and could be lower [2]. Our patient had a low total leucocyte count (TLC) (2.9 × 10^9^/L) which made the diagnosis of DS all the more difficult. Imaging proved to be crucial in this regard.

Here we discuss the case of a 14-year-old Indian girl who came to our hospital for treatment of APML and subsequently developed symptoms which later came to be diagnosed as DS based on the initial clinical presentation on starting induction with ATRA and ATO, subsequent stormy clinical course, quick response to withdrawal of ATRA and ATO, specific treatment with steroid and resolution of radiological findings.

## Case presentation

**Fig.1:**
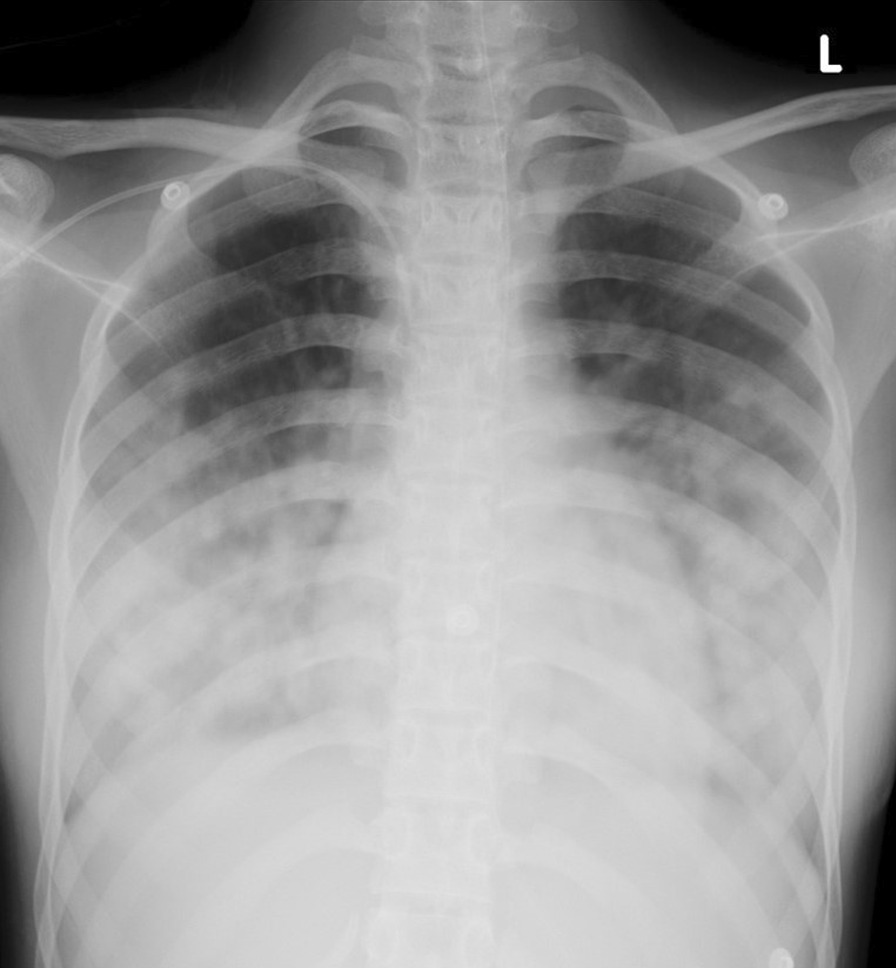
Chest radiograph showing bilateral middle and lower zone alveolar opacities with sparing of upper zones

A 14-year-old Indian girl presented with a history of sudden onset shortness of breath and was sent to the radiology department for a chest radiograph (CXR). The image showed bilateral middle and lower zone pulmonary alveolar infiltrates with relative sparing of the upper zones (Fig. 1). Her medical history indicated that she had been diagnosed with APML and had been started on chemotherapy as per the International Consortium for Childhood APML 02 protocol which included ATO and ATRA. Table 1 shows her initial laboratory results at admission. She developed worsening respiratory distress soon after the initiation of chemotherapy with increasing oxygen dependence and was transferred to the Pediatric Intensive Care Unit for ongoing care. ATRA and ATO were discontinued. Table 2 shows her laboratory test results after she became symptomatic.

**Table 1:** Initial laboratory test results at admission

Parameter	Values
Haemoglobin	6.5 g%
Total Leucocyte count	6.7 x 10^9^/L
Erythrocyte sedimentation rate)	62 mm in 1st hour
Platelet count	1.85 × 10^9^/L

**Table 2:** Laboratory results after patient became symptomatic

Parameter	Value
Haemoglobin	3.7g%
Total leucocyte count	2.9 × 10^9^/L
Erythrocyte sedimentation rate	62 mm in first hour
Platelet count	0.07 × 10^9^/L

**Fig.2:**
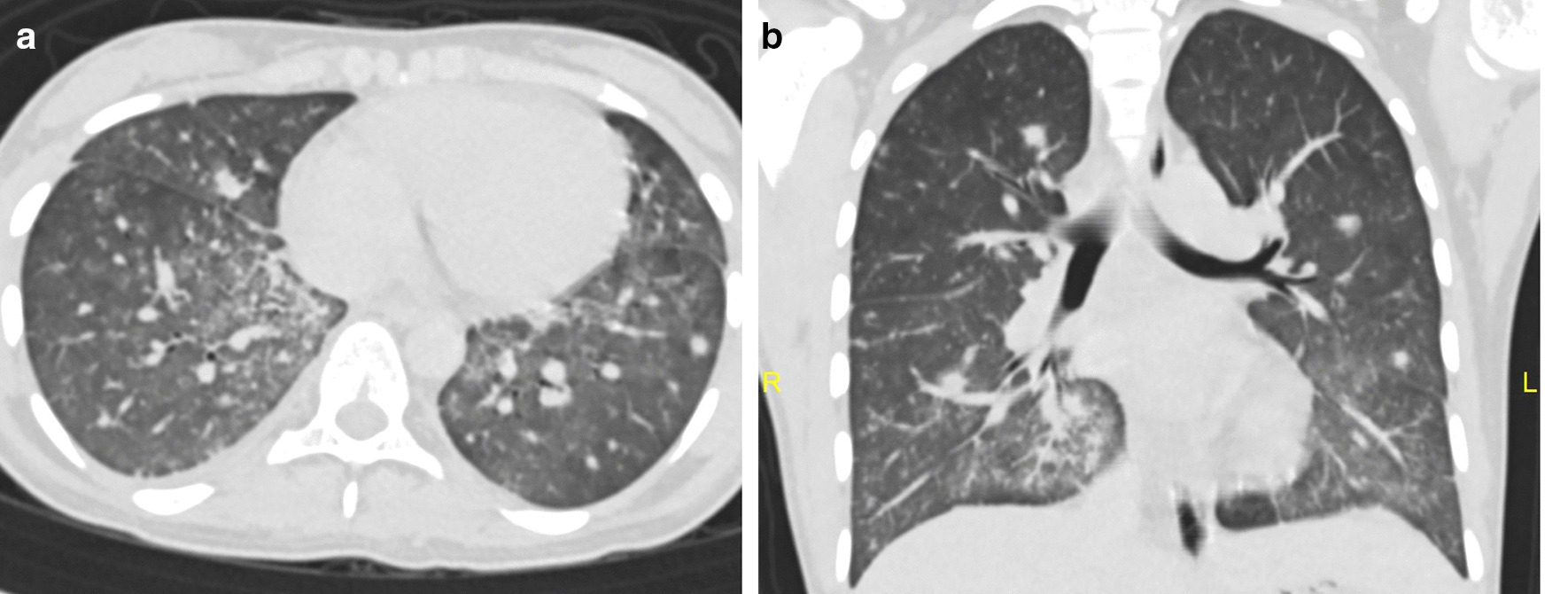
**a** Axial computed tomography (CT) scan showing bilateral pulmonary parenchymal nodules and ground-glass changes in the middle lobe, lingular segment of left upper lobe and bilateral lower lobes. Upper lobes are less affected. **b** Coronal CT scan showing bilateral pulmonary parenchymal nodules and ground-glass changes in the middle lobe, lingular segment of left upper lobe and bilateral lower lobes. Upper lobes are less affected

Without any further testing to avoid wasting valuable time, systemic steroid (dexamethasone 10 mg/m^2^) therapy was initiated. Appropriate respiratory care in the form of bilevel positive airway pressure (BiPAP) was given. A computed tomography (CT) scan of thorax showed multiple bilateral lung nodules with surrounding changes in ground-glass opacity predominantly in the lower lobes (Fig. 2).The differential diagnosis based on her imaging findings included infective causes (bacterial, viral, fungal), leukemic infiltrates, acute respiratory distress syndrome (ARDS), leukostasis as part of hyperleukocytosis, drug toxicity, pulmonary oedema and pulmonary haemorrhage. Leukostasis was ruled out since the patient had a low TLC (2.9 × 10^9^/L), and a requirement for leukostasis is a very high WBC count (> 100 × 10^9^/L). Further, radiological studies revealed lung nodules with patchy changes in ground-glass opacity whereas leukostasis is characterised by more confluent opacities with interlobular septal thickening.

**Fig. 3:**
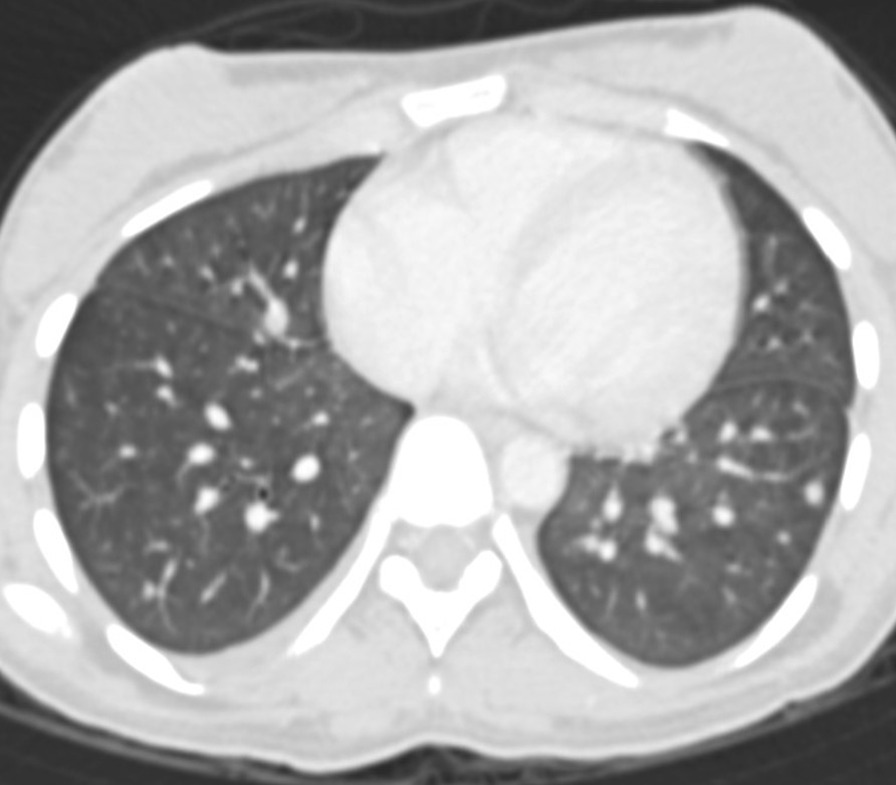
Follow-up axial computed tomography scan showing a significant reduction of pulmonary nodules and ground-glass changes in the middle lobe, lingular segment of left upper lobe, and bilateral lower lobes

**Fig. 4:**
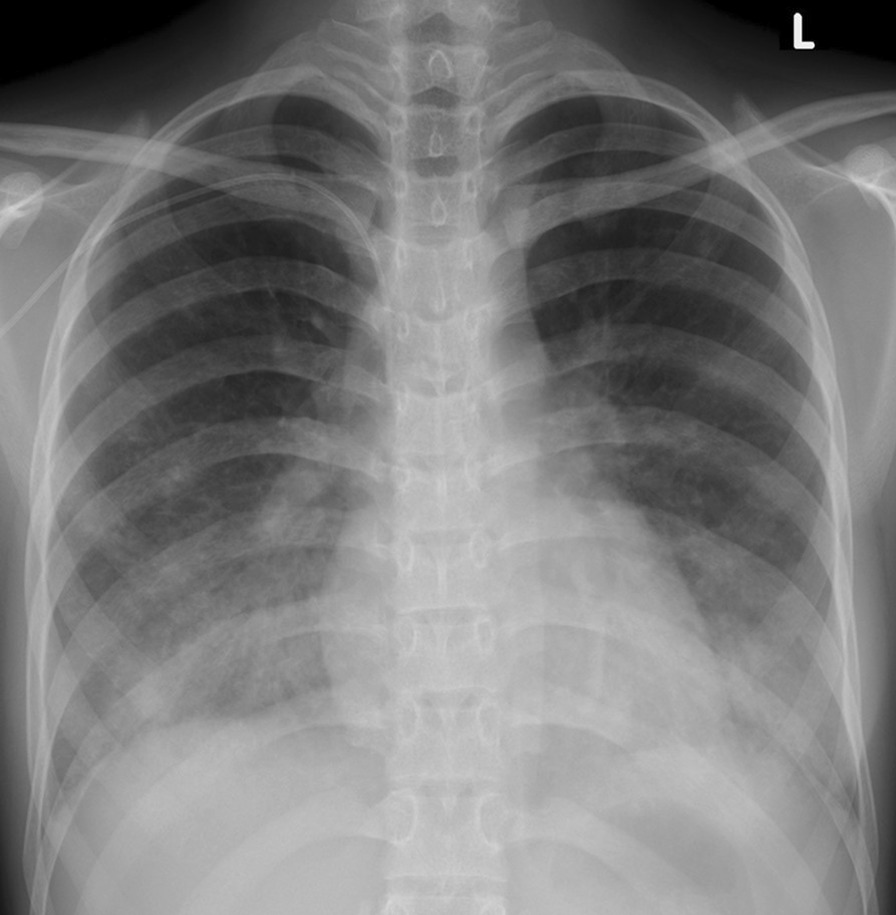
Follow-up chest radiograph showing a significant reduction of pulmonary alveolar opacities previously seen in bilateral middle and lower zones

With the treatment described above, her clinical, in particular her respiratory status, improved rapidly. A subsequent CT scan of the abdomen after 4 days of treatment showed a reduction in the lung changes at the visualised lung bases (Fig. 3). The changes in ground-glass opacity had almost disappeared with subsidence of the nodules. A CXR performed at around the same time also showed a reduction in the lung changes (Fig. 4), with bilateral reduction of the infiltrates in the middle and lower zones. Based on the clinical improvement and imaging changes following withdrawal of ATRA/ATO and starting of dexamethasone, the diagnosis that met all the features was that of differentiation syndrome. This was a rther unique diagnosis since most cases of DS reported in the literature are usually associated with high TLC (> 5 × 10^9^/L). Thus, based on the radiological features and clinical correlation a difficult yet crucial diagnosis was possible which significantly affected the patient’s further treatment.

## Discussion and conclusions

Differentiation syndrome (originally called “retinoic acid syndrome”) is a potentially fatal complication that can arise during the treatment of APML with ATRA and/or ATO.

The reported incidence of DS in patients with APML treated with standard doses of ATRA ranges from 2 to 27%, compared to 7–31% in patients treated with ATO [4]. De Botton et al. [5] reported in a large series of patients with DS that respiratory signs and symptoms and fever were the most frequent clinical presentations of DS. These authors described 413 patients with newly diagnosed APML, 64 (15%) of whom developed DS (median at day 7), with nine (14%) patients dying from it [5].

The most common manifestations of DS are pulmonary infiltrate, pleural effusion and respiratory distress. Cardiac involvement is more commonly characterised by pericardial effusion, but it can also present as chest pain typical of coronary obstruction. Rarer clinical presentations, such as musculoskeletal symptoms, have also been reported. There is no pathognomonic clinical sign or laboratory test to diagnose DS. For this reason, DS can sometimes be misdiagnosed or confounded with other concurrent medical conditions, such as infection and heart failure. It has been suggested that DS be considered when at least three of the following signs, symptoms or imaging features are present: fever, weight gain, respiratory distress, pulmonary infiltrates, pleural or pericardial effusions, hypotension and renal failure [3].

The pathogenesis of APML-linked DS is complex and not well understood. In essence, ATRA is thought to: (1) lead to a release of a variety of cytokines by differentiating blast cells and (2) induce a change in adhesive properties on blast cells. Pro-inflammatory cytokines, including interleukin 1 beta (IL1B), IL6, IL8 and tumour necrosis factor alpha are released, which leads to a systemic inflammatory response syndrome (SIRS). SIRS manifests as fever, tachycardia and tachypnoea and can progress into shock if left untreated. The release of cathepsin G increases vascular permeability and causes endothelial damage [2].

Univariate analysis identified a high WBC count at presentation, with 5 × 10^9^/L as the most significant cutoff point, as a prognostic factor for severe DS [6]. This needs to be noted since in our case the TLC was much lower (2.9 × 10^9^/L). Other prognostic factors for severe DS are abnormal levels of serum creatinine; FLT3-Internal Tandem Duplications (ITD) mutations; microgranular French American British (FAB) subtype; short promyelocytic leukaemia-retinoic acid receptor alpha (PML-RARA) isoform; and male sex [6]. As already stated, there are no clinical signs or laboratory tests to diagnose DS, nor is there a radiological finding pathognomonic for DS. Radiological features may be explained by the proposed hypotheses of pathophysiology of the DS. Most of the patients with DS show cardiomegaly, widening of the vascular pedicle width, increased pulmonary blood volume, peribronchial cuff, ground-glass opacity, septal lines and pleural effusion: these findings are similar to those of congestive heart failure with pulmonary oedema, but they can also probably be produced by leukemic lung infiltration and endothelial leakage [7]. In mild DS, lesions are prevalent in the lower lobes, while in severe DS, the lesions are diffuse, with no difference between peripheral or central regions [7]. Davis et al. [8] reported CT findings in three patients with DS, consisting of peripheral nodules, reticular and ground-glass opacity and pleural effusions. The CT scan showed similar findings in our case. These authors also reported the case of a patient with DS who developed pneumothorax [8]. Other reported histological findings in analyses of lungs are extensive interstitial and alveolar lung infiltration by maturing myeloid cells, endothelial cell damage, oedema, haemorrhage and fibrinous exudates that correspond to poorly defined centrilobular nodules and ground-glass opacity with or without interlobular septal thickening [7].

Patients with APML and DS have a highly compromised immune system; therefore, it is not a rare event to find other concurrent pathologic conditions, such as pneumonia or fungal infections, which can confound the radiological picture of DS syndrome [7]. In our case also, the differential diagnosis was fungal infection. Hence, the treating physicians need to be aware of the clinical context to raise the possibility of DS when these findings are seen on CXR or CT, which our case further stressed. The low TLC makes our case different since most of the cases of DS reported to date had a high TLC.

As DS can have a subtle clinical picture at presentation but progress rapidly, it is of utmost importance to be aware of this complication and initiate therapy as soon as this syndrome is suspected. Dexamethasone is considered to be the mainstay of treatment of DS and should be administered at the first sign or symptom of this syndrome [3].

The same protocol was followed in our case after the withdrawal of ATRA/ATO. Gradually, once the patient responded to treatment, as was seen in our patient, the chemotherapy regimen could be restarted [3]. The strength of this report is that it paves the way for widening our range of differentials in the future for patients with APML who develop such symptoms. The main limitation is that the imaging features were not specific to the cause and there is no pathognomic test for clinching the diagnosis, as stated earlier.

## Conclusion

Differentiation syndrome is a rare but severe complication of APML that can develop in patients treated with ATRA/ATO. The imaging features observed on CXR or CT scan of thorax have a wide range of differentials. Based on this case report, it is evident that DS should always be kept in mind. Early diagnosis can help expedite the treatment of the patient, thereby increasing chances of survival.

## Data Availability

Not applicable.
